# Phylogeographic study of the *Bufo gargarizans* species complex, with emphasis on Northeast Asia

**DOI:** 10.1080/19768354.2021.2015438

**Published:** 2021-12-20

**Authors:** Changhoon Lee, Jonathan J. Fong, Jian-Ping Jiang, Pi-Peng Li, Bruce Waldman, Jong Ryol Chong, Hang Lee, Mi-Sook Min

**Affiliations:** a Conservation Genome Resource Bank for Korean Wildlife, Research Institute for Veterinary Science and College of Veterinary Medicine, Seoul National University, Seoul, South Korea; b Team of Climate Change Research, National Institute of Ecology, Seocheon-gun, South Korea; c Science Unit, Lingnan University, Tuen Mun, Hong Kong, People’s Republic of China; d CAS Key Laboratory of Mountain Ecological Restoration and Bioresource Utilization & Ecological Restoration Biodiversity Conservation Key Laboratory of Sichuan Province, Chengdu Institute of Biology, Chinese Academy of Sciences, Chengdu, People’s Republic of China; e Center for Chinese Endemic Herp-Breeding and Conservation Research and Liaoning Key Laboratory of Evolution and Biodiversity, Shenyang Normal University, Shenyang, People’s Republic of China; f Department of Integrative Biology, Oklahoma State University, Stillwater, OK, USA; g Wildlife Research Center, Korea University, Tokyo, Japan

**Keywords:** Amphibian, *Bufo gargarizans*, phylogeny, Northeast Asia, Korean Peninsula, pleistocene, glacial refugia

## Abstract

We conduct a phylogeographic and population genetic study of the Asiatic toad (*Bufo gargarizans*) to understand its evolutionary history, and the influence of geology and climate. A total of 292 individuals from 94 locations were genotyped for two mitochondrial loci (*cytb*, *ND2*) and five nuclear introns (*Sox9-2*, *Rho-3*, *CCNB2-3*, *UCH-2*, and *DBI-2*), and we performed a suite of phylogenetic, population genetic, and divergence dating analyses. The phylogenetic trees constructed using mitochondrial loci inferred *B. gargarizans* being divided into two major groups: China mainland and Northeast Asia (Northeast China, Russia, and Korean Peninsula). As with previous studies of this species, we recover population genetic structure not tied to geographic region. Additionally, we discover a new genetic clade restricted to Northeast Asia that points towards the Korean Peninsula being a glacial refugium during the Pleistocene. The weak phylogeographic pattern of *B. gargarizans* is likely the result of multiple biological, anthropogenic, and historical factors – robust dispersal abilities as a consequence of physiological adaptations, human translocation, geologic activity, and glacial cycles of the Pleistocene. We highlight the complex geologic and climatic history of Northeast Asia and encourage further research to understand its impact on the biodiversity in the region.

## 1. Introduction

### 1.1. Bufo gargarizans species complex

The Asiatic toad, *Bufo gargarizans*, (Cantor 1842), is distributed in China, Russia, North Korea, South Korea, and Japan (Miyako and Ryukyu Islands). *Bufo gargarizans* is currently classified as Least Concern in the IUCN Red List because of its large habitat range, but it is likely to be classified as a risk category if a threat occurs (IUCN 2019). This species has had a complicated taxonomic history (Hu et al. 1984; Huang et al. 1990; Macey et al. 1998; Liang et al. 2010), with several species being recognized as distinct or the same species as *B. gargarizans* (Fu et al. 2005). Some molecular studies considered *B. andrewsi*, *B. minshanicus*, and *B. tibetanus* to be synonyms of *B. gargarizans*, while *B. bankorensis* is treated as a distinct species (Chen et al. 2013; Yu et al. 2014; Tong and Wo 2017; Frost 2020).

Genetic studies of *B. gargarizans* have provided insight into its evolutionary history. An allozyme-based study of *B. gargarizans* identified clear genetic difference between individuals from Korea and China (Yang et al. 2000). Zhan and Fu (2011) performed a multilocus molecular study focused on China using both mitochondrial DNA (mtDNA) and nuclear DNA (nuDNA), and sampled *B. gargarizans* covering four major geographic regions in China (West [W], Central [C], Southeast [SE], and Northeast [NE]); they recovered seven clades not differentiated based on geography (e.g. Clade A included samples from the W, C, and NE). Borzée et al. (2017) extended this work by identifying a distinct clade of individuals from South Korea and across China. Although Borzée et al. (2017) broadened geographic sampling by including Korea, they did not include the dataset of Zhan and Fu (2011) and only collected mtDNA data (control region and associated tRNAs, and NADH dehydrogenase 2 [*ND2*]). Zhan and Fu (2011) and Borzée et al. (2017) contributed to our understanding of the evolutionary history of *B. gargarizans*, but both had reasonable limitations in their study designs (e.g. limited sampling, limited data types). We combine the strengths of these previous studies by including comprehensive geographic sampling and multiple molecular loci.

### 1.2. Phylogeography of Northeast Asia

Northeast Asia (Korean Peninsula, China, and Russia) has had a complex geological and climatic history, which affected the evolution and dispersal of organisms in the region. During the Pleistocene, major geological events (e.g. the opening of the Yellow Sea, forming of major mountain ranges) and dramatic climate change are believed to have affected the distribution of terrestrial vertebrates (Lee et al. 2008; Zhang et al. 2008; Sakka et al. 2010; Zhang et al. 2012; Kim et al. 2013; Koh et al. 2013; Fong et al. 2016; Borzée et al. 2017; Park et al. 2019; Fong et al. 2020). In particular, studies confirmed that the Korean Peninsula was not covered by glaciers during the latest glacial cycle (Kong 2000; Yi and Kim 2010), and this region played an important role as a refugium in preserving genetic diversity (Lee et al. 2008; Zhang et al. 2008; Kim et al. 2013; Fong et al. 2020). Research results to preserve biodiversity, such as acoustic analysis studies in this area (Choi et al. 2019), emphasize the importance of this area.

Studies on extant amphibians in this region have clarified the phylogeography of the Chinese black-spotted frog (*Pelophylax nigromaculatus*) (Zhang et al. 2008), Oriental fire-bellied toad (*Bombina orientalis*) (Fong et al. 2016), Japanese tree frog (*Dryophytes japonica* group) (Dufresnes et al. 2016), brown frog (*Rana dybowskii*) (Yang et al. 2017), water toad (*B. stejnegeri*) (Fong et al. 2020), and Asiatic toad (*B. gargarizans*) (Zhan and Fu 2011; Borzée et al. 2017). Each study had species-specific goals, but a common finding was that there is genetic divergence between China and Korea likely due to geology (mountain and oceanic barriers) and Pleistocene glacial cycles. For *B. gargarizans*, Zhan and Fu (2011) inferred western China to be a major refugium for *B. gargarizans* owing to high genetic diversity in the region, while Borzée et al. (2017) inferred that this species dispersed westward from the Korean Peninsula across land bridges during low sea levels. We provide more clarity on the evolution and dispersal process of *B. gargarizans* in Northeast Asia.

### 1.3. Goals

Despite being the focus of several studies, the evolutionary history of *B. gargarizans* remains uncertain. Our study clarifies the phylogeography of *B. gargarizans* as a basis for understanding its historical dispersal patterns in relation to the geologic and climatic history of the region. Our study improves on previous studies by increasing geographic coverage by adding samples from the Northeast Asia (North Korea, South Korea, and Russia), as well as sampling multiple genetic loci.

## 2. Materials and methods

### 2.1. Study areas

**Figure 1.**
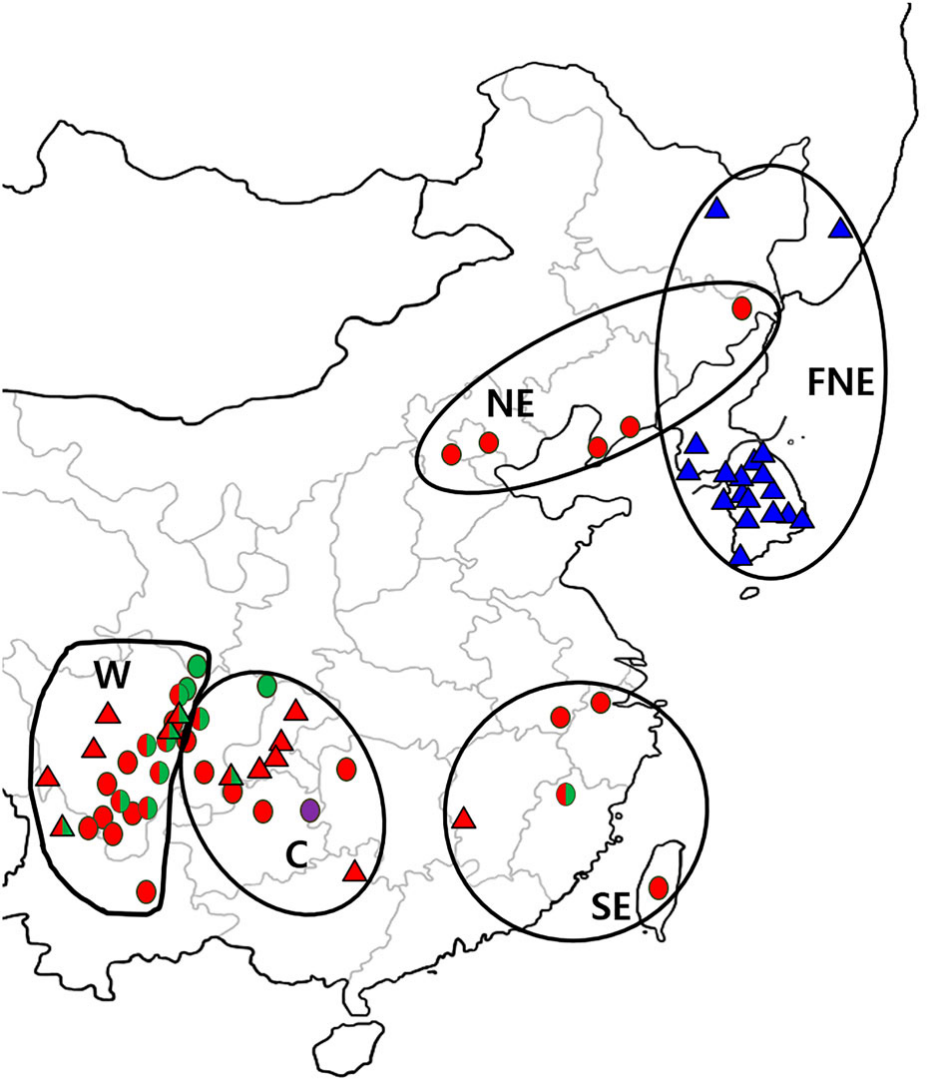
Sampling localities of specimens used in this study. Triangles are samples that are new from this study. The phylogenetic analysis identified seven major clades, which we indicate in this map (red = clades A, B, C, D; purple = clade E; green = clades F, G; blue = clade H). Samples are also separated based on region following Zhan and Fu (2011) (West [W], Central [C], Southeast [SE], Northeast [NE]) and a new region identified on our study (Far Northeast [FNE]). Colors and regional category correspond with Figure 2.

Genetic data were collected from 292 *B. gargarizans* individuals from four countries (China, North Korea, South Korea, and Russia). Among them, 165 Chinese samples were from GenBank (Zhan and Fu 2011), while the remaining 127 were newly sequenced in this study. Of these new samples, 87 individuals were from South Korea, three from North Korea, three from Russia, and 34 from China (Figure 1; Table S1). Following Zhan and Fu (2011), we categorized samples a priori based on geography (W, C, SE, NE), while adding a new region based on our study called Far Northeast ([FNE]: Korea, Russia, and Northeast China). Tissue samples (toe tips, muscle, or tadpole tails) of new specimens were collected from the field and preserved in 95% ethanol.

### 2.2. Laboratory methods

Genomic DNA was extracted from tissue samples using the DNeasy Blood & Tissue Kit (Qiagen, Venlo, Netherlands) following the manufacturer's protocols. Seven molecular markers were amplified and sequenced in this study: two mtDNA (cytochrome b [*cytb*], *ND2*) and five nuDNA (rhodopsin intron 3 [*Rho-3*], sex determining region Y box containing gene 9 intron 2 [*Sox9-2*], cyclin B2 intron 3 [*CCNB2-3*], diazepam binding inhibitor intron 2 [*DBI-2*], and ubiquitin carboxyl-terminal hydrolase intron 2 [*UCH-2*]). Polymerase chain reaction (PCR) was performed in 30 μL reactions with 3 μL 10X Buffer (1X/μL), 2.4 μL dNTP (0.2 pm/μL), 1.5 μL each primer (0.5 pm/μL), 0.2 μL Taq polymerase (1U/μL), 20.4 μL distilled water, and 1 μL template DNA (10 ng/μL). The 10X Buffer, dNTP, and Taq polymerase were from the i-star Taq^TM^ DNA polymerase kit (iNtRON Biotechnology, Seongnam, Gyeonggi, Korea). Detailed PCR conditions for each marker are in Table S3, and primer information is in Table S4.

The size of PCR products was confirmed with electrophoresis on 1% agarose gels. When multiple bands were found on the gel, PCR products were run again on a 2% agarose gel and the correct-sized band was excised. The PCR products and gel fragments were purified using DNA purification columns (Zymo Research, Irvine, CA, USA). Sequencing was performed in both directions using the PCR primers on an Applied Biosystems 3730XL machine and Big Dye® Terminator v3.1 Cycle Sequencing Kit (Applied Biosystems, Foster City, CA, USA) at the National Instrumentation Center for Environmental Management of Seoul National University (Seoul, Korea). Due to the large size of *cytb* (>1,000bp), an additional primer (Bufo3-Inner1) was used for sequencing (Fong et al. 2020; Table S4).

### 2.3. Phylogenetic analyses

Consensus sequences were created by assembling the forward and reverse sequences using Geneious Pro v.5.3.6 (Biomatters, Auckland, New Zealand). Multiple sequence alignments were performed using Clustal X (Larkin et al. 2007). Haplotype sequences of *Bufo* species obtained in this study were deposited in GenBank (Table S2). Genetic distances within and between species were calculated with MEGA v5.2 (Tamura et al. 2007).

Phylogenetic analyses were performed on the seven individual gene datasets (*cytb*, *ND2*, *Rho-3*, *Sox9-2*, *CCNB2-3*, *DBI-2*, and *UCH-2*), and a concatenated dataset (all genes except *cytb*) (Table S1). *Cytb* was not included in the combined dataset to enable comparison with Zhan and Fu (2011). A selection of 1–4 outgroups (*B. bufo*, *B. stejnegeri*, and *B. japonicus* [two individuals]) was used for the individual analyses (Table S2). For the individual gene datasets, both maximum likelihood (ML) and Bayesian inference (BI) analyses were performed. The ML analyses were conducted using the combined ML search and rapid bootstrap in RAxML v.8.0.2 (Stamatakis 2014). A GTR + G model of sequence evolution was used for the ML tree search with 1,000 bootstrap replicates. BI analyses were performed using MrBayes v.3.2 (Ronquist et al. 2012) by running four chains for 2 million generations, sampling every 1,000th generation. The best fit model of evolution was estimated based on the Bayesian Information Criterion (BIC) in jModeltest v.1.0 (Table S5) (Guindon and Gascuel 2003; Posada 2008). All analyses were conducted using the CIPRES Science Gateway (Miller et al. 2010). Trees were viewed and edited using FigTree v.1.4.0 (Rambaut 2012).

For the concatenated dataset, POFAD (Joly and Bruneau 2006) was used to construct a multilocus phylogeny. The POFAD (for Phylogeny of Organisms from Allelic Data) algorithm combines genetic distance matrices generated from allelic data of individual loci into a single genetic-distance matrix (Joly and Bruneau 2006). The analysis included 79 individuals (68 from Zhan and Fu [2011] and 11 from this study). The uncorrected pairwise distances of each marker were generated in MEGA v.5.2 (Tamura et al. 2007), then POFAD was used to obtain a standardized combined-locus distance matrix. Subsequently, a neighbor joining (NJ) phylogenetic tree was reconstructed based on the combined genetic-distance matrix using MEGA v.5.2 (Tamura et al. 2007).

### 2.4. B. gargarizans in the Northeast Asia

We performed additional analyses focusing on samples from Northeast Asia. To examine the relationships between samples and allow for reticulation, a haplotype network was built for the *ND2* dataset using HapStar v.0.7 (Teacher and Griffiths 2011). The median-joining network was constructed using Network v.5.0.1. (Bandelt et al. 1999, fluxus-engineering.com). NuDNA was not included because these data showed low variation and poor resolution. Population genetic analyses were performed on the same *ND2* dataset. The parameters calculated were the number of haplotypes, haplotype diversity (ℎ), and nucleotide diversity (π) (Nei 1987) using DnaSP v5.0 (Librado and Rozas 2009). Additionally, we conducted an analysis of molecular variance (AMOVA) to determine the hierarchical genetic structure among populations using Arlequin v.3.5 (Excoffier and Lischer 2010).

### 2.5. Divergence time estimation within B. gargarizans

We estimated the divergence times between the major clades of *B. gargarizans*. Divergence dating analyses were performed using BEAST v.1.7.5 (Drummond et al. 2012) on the *cytb* dataset, as data from other species of the Bufonidae were publicly available (Recuero et al. 2012). We were unable to include *B. gargarizans* sampling from Zhan and Fu (2011), as they did not collect *cytb* data. We focused on the divergence dating results for the major *B. gargarizans* clades (A, B, G, and H), and within Clade H (Korea, Northeast China, and Russia). Five species were included as outgroups from our own data (*B. stejnegeri* and *B. japonicus*) and GenBank (*B. tibetanus*, *B. melanostictus*, and *Telmatobius bolivianus*) (Table S2).

For two calibration points based on fossils, we followed Recuero et al. (2012) by setting a prior distribution for the root of the *B. bufo* group (*B. bufo*, *B. eichwaldi*, *B. spinosus*, and *B. verrucosissimus*) (lognormal distribution with an offset of 9.7 mega-annum (Ma), and 95% of the values between 10.1 and 22.2 Ma) and for *B. verrucosissimus* (lognormal distribution with an offset of 1.81 Ma and 95% of the values between 2 and 4.5 Ma). The birth–death process was speciﬁed for the tree prior since it is well suited for a multi-species dataset with deep genetic divergence across clades and species. Two independent runs of 100 million generations, sampling every 10 thousand generations, were combined after checking for convergence and adequate effective sample sizes (ESSs) of parameters using the software Tracer v.1.7.1 (Rambaut et al. 2018).

## 3. Results

### 3.1. Phylogenetics and population structure of B. gargarizans

**Figure 2.**
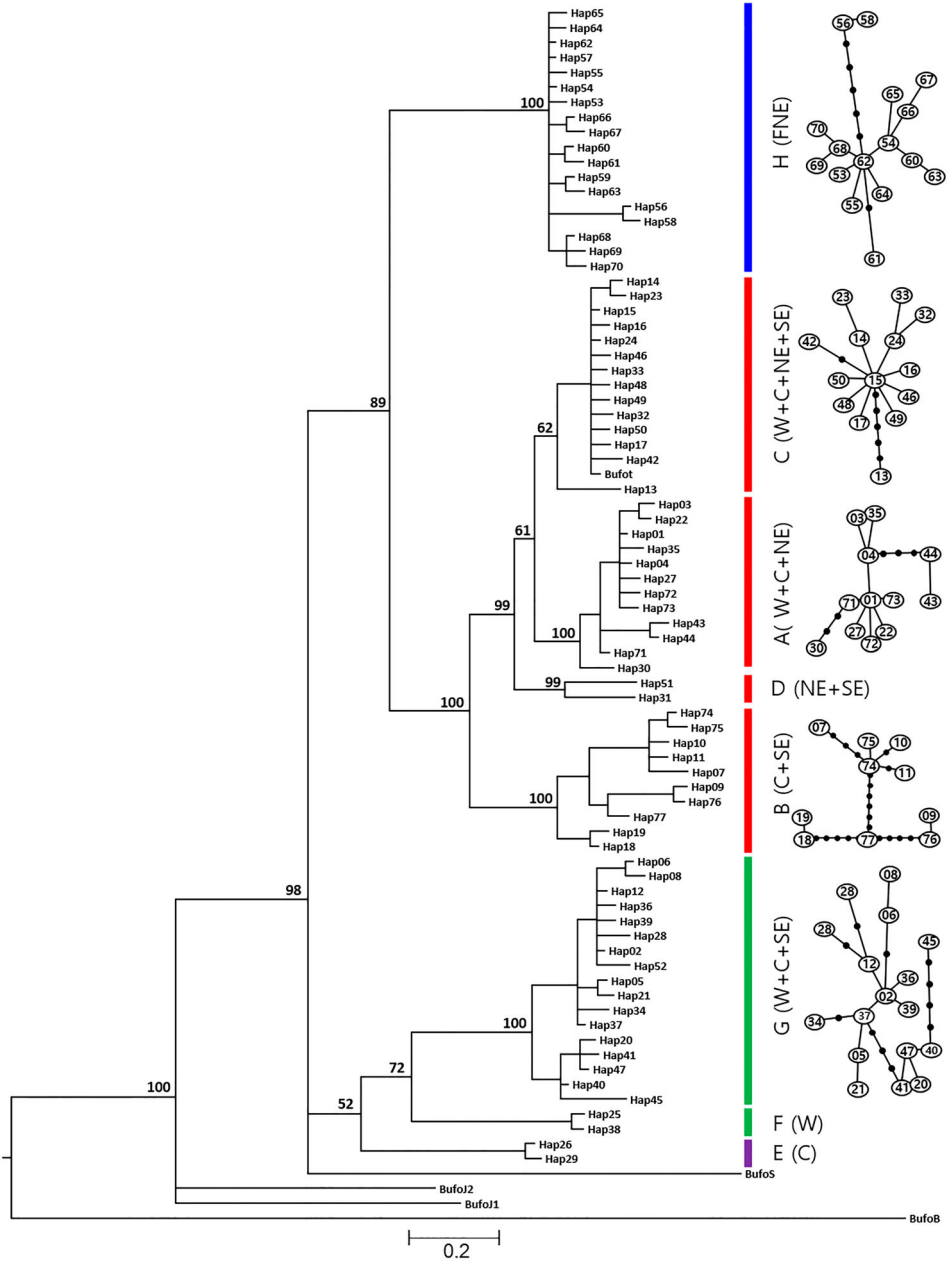
Bayesian phylogenetic tree based on NADH dehydrogenase 2 (*ND2*) haplotype dataset generated using TCS. Numbers above branches are Bayesian posterior probabilities of the major clades. Haplotype names follow Table S1. Colored bars indicate the major clades (red = clades A, B, C, D; purple = clade E; green = clades F, G; blue = clade H). For each of these clades, the origin of the samples is indicated in parentheses: West (W), Central (C), Southeast (SE), Northeast (NE). Colors and regional category correspond with Figure 1.

The phylogenetic trees for the five nuclear loci are in the supplementary materials (Figures S1–S5). We focus on the *ND2* dataset, as it had the greatest resolution. For *ND2*, 77 haplotypes were detected for *B. gargarizans*, with a mean intraspecific pairwise distance of 0.0535 (0.0018–0.0722) (Table 3). There were 153 polymorphic sites, of which 63 were singleton-variable and 90 were parsimony-informative sites. The BI analysis recovered eight clades (Figure 2) which was similar to the results from the ML analysis. We label the clades A–G, following Zhan and Fu (2011), while identifying a new clade (Clade H) containing samples exclusive to Northeast Asia (Korean Peninsula, Northeast China [Heilongjiang Province], and Russia). For intra-clade genetic distances, the highest values were in clade G, while the lowest values were in clade A (Table 1). Clade H contained 18 haplotypes (Table 2). Pairwise distances between the Clade H and the other seven clades ranged between 0.0451–0.0722 (Table 1).

**Table 1.** Average genetic distances between the major clades of *B. gargarizans*, based on the NADH dehydrogenase 2 (*ND2*) dataset.

	A	B	C	D	E	F	G	H
A	-	-	-	-	-	-	-	-
B	0.0380	-	-	-	-	-	-	-
C	0.0200	0.0383	-	-	-	-	-	-
D	0.0253	0.0439	0.0263	-	-	-	-	-
E	0.0544	0.0569	0.0539	0.0613	-	-	-	-
F	0.0629	0.0692	0.0569	0.0646	0.0529	-	-	-
G	0.0668	0.0707	0.0603	0.0694	0.0531	0.0460	-	-
H	0.0451	0.0576	0.0527	0.0533	0.0625	0.0635	0.0722	-

**Table 2.** Information for the eight major clades based on the NADH dehydrogenase 2 (*ND2*) dataset. Region refers to the presence (▪) or absence (-) of samples from the five major geographic regions.

ND2 Clade	Number of Haplotypes	Region				
		West	Central	Southeast	Northeast	Far Northeast
A	12	▪	▪	-	▪	-
B	10	-	▪	▪	-	-
C	15	▪	▪	▪	▪	-
D	2	-	-	▪	▪	-
E	2	-	▪	-	-	-
F	2	▪	-	-	-	-
G	17	▪	▪	▪	-	-
H	18	-	-	-	-	▪

Haplotypes of individuals from the four geographic regions identified by Zhan and Fu (2011) were found in multiple genetic clades: W region had haplotypes in four genetic clades (A, C, F, and G), with clade F being exclusive to this region; C region had haplotypes in five genetic clades (A, B, C, E, and G), with clade E being exclusive to this region; the SE region had haplotypes in four genetic clades (B, C, D, and G); and the NE region had haplotypes in three genetic clades (A, C, and D) (Table 2). FNE, the new region delineated in our study, had haplotypes exclusive to Clade H (Table 2).

Genetic diversity indices are summarized in Table 3. The number of singleton variable site (S) was 32 and nucleotide diversity (π) was 0.04412. Areas with high genetic diversity indices were the W region (68 polymorphic sites, 49 parsimony-generic sites, 19 single-variable sites) and the C region (nucleotide diversity = 0.03793). Pairwise distances and nucleotide diversity were lowest in the FNE region (Table 3).

**Table 3.** Molecular diversity of the five major geographic regions.

Region	n	*p*	*P*	S	Pi	π
West	36	5.2% (0.2–6.7%)	68	19	49	0.03636
Central	17	5.1% (0.2–7.1%)	57	9	48	0.03793
Southeast	15	5.1% (0.26–7.1%)	57	12	45	0.03488
Northeast	5	2.0% (0.2–3.1%)	21	13	8	0.01938
Far Northeast	18	0.7% (0.1–2.0%)	20	10	10	0.00725
Overall	77	5.4% (0.2–7.2%)	109	32	77	0.04412

n: number of haplotypes, *p*: pairwise difference, *P*: polymorphic site, S: singleton variable site, Pi: parsimony informative site, π: nucleotide diversity

**Figure 3.**
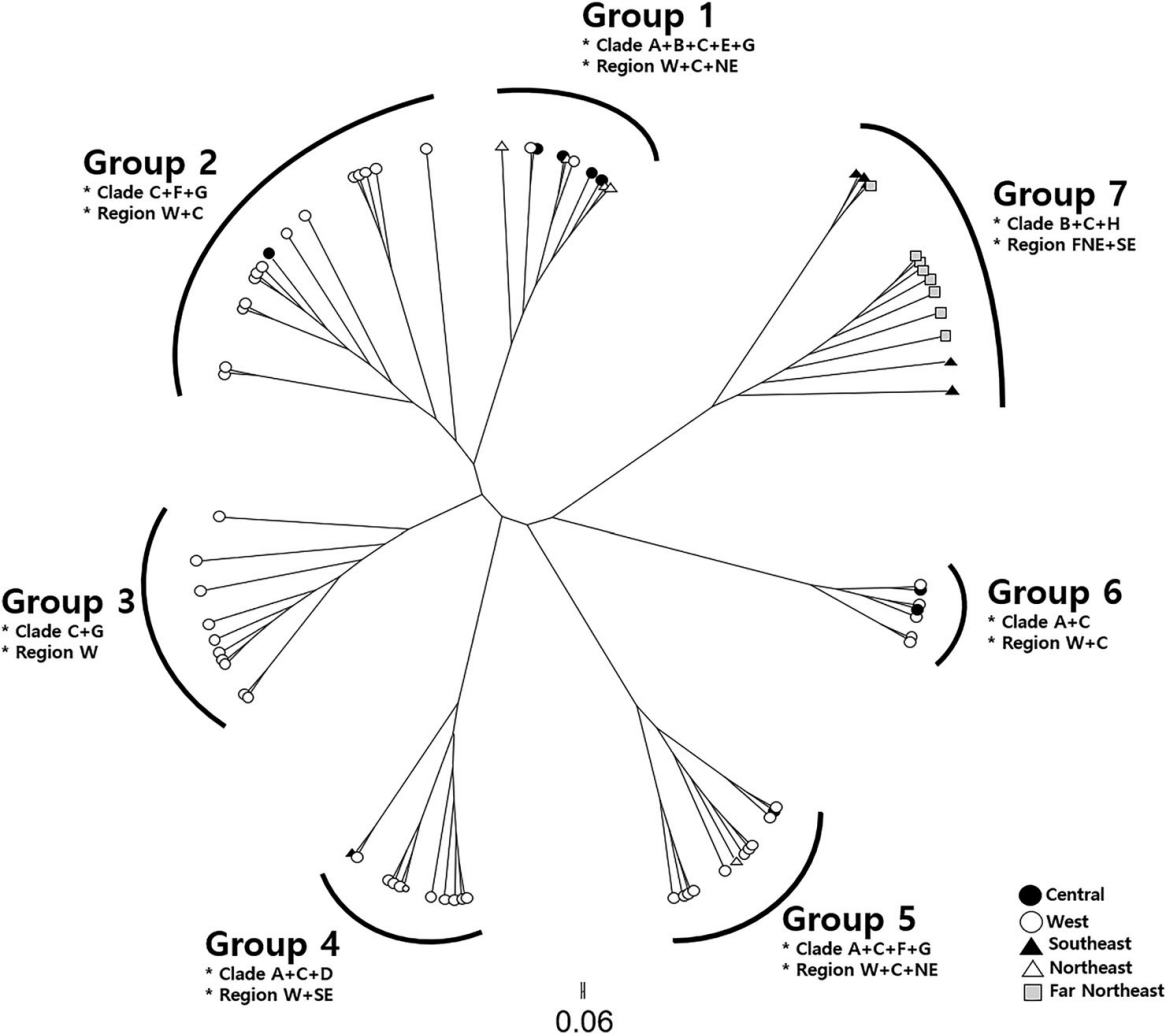
Neighbor-joining phylogram based on a concatenated dataset of six genetic markers (mtDNA *ND2*, nuDNA *Rho-3*, *Sox9-2*, *CCNB2-3*, *DBI-2*, *UCH-2*) analyzed using POFAD. This analysis separated the samples into seven genetically distinct groups. The categorization of individuals based on geographic region is indicated by different shapes/color: West (white circle), Central (black circle), Northeast (white triangle), Southeast (black triangle), Far Northeast (gray square). Clade and region information refer to Figures 1 and 2.

The multilocus NJ phylogram from POFAD recovered seven major groups, with one being unique to our study (Group 7) (Figure 3). The POFAD algorithm implements confrontational information to infer the genetic distance between individual. The algorithm differs from the phylogenetic analysis in that it constructs and visualizes comparative data in the network by calculating the interactive distance matrix of the combined genetic information of each sample, then averaging the distances between the haploids. The clade composition for each geographic group is shown in the BI analysis (Figure 2), confirming that some geographic groups are distributed across multiple clades. For the six groups found in the NJ phylogram of Zhan and Fu (2011), there was no correlation with either region or altitude, with individuals from the W region included in all groups. Members of the newly identified Group 7 in this study (Figure 3) came from the SE region (Fujian and Zhejiang Provinces in China) and the FNE region (Korea, Russia, and Heilongjiang Province of China).

Bayesian tree for each nuDNA (*Rho-3*, *Sox9-2*, *CCNB2-3*, *DBI-2*, *UCH-2*) was analyzed, however, the results of the five nuDNAs analysis could not be interpreted (Figure S 1–5).

### 3.2. B. gargarizans in Northeast Asia

**Figure 4.**
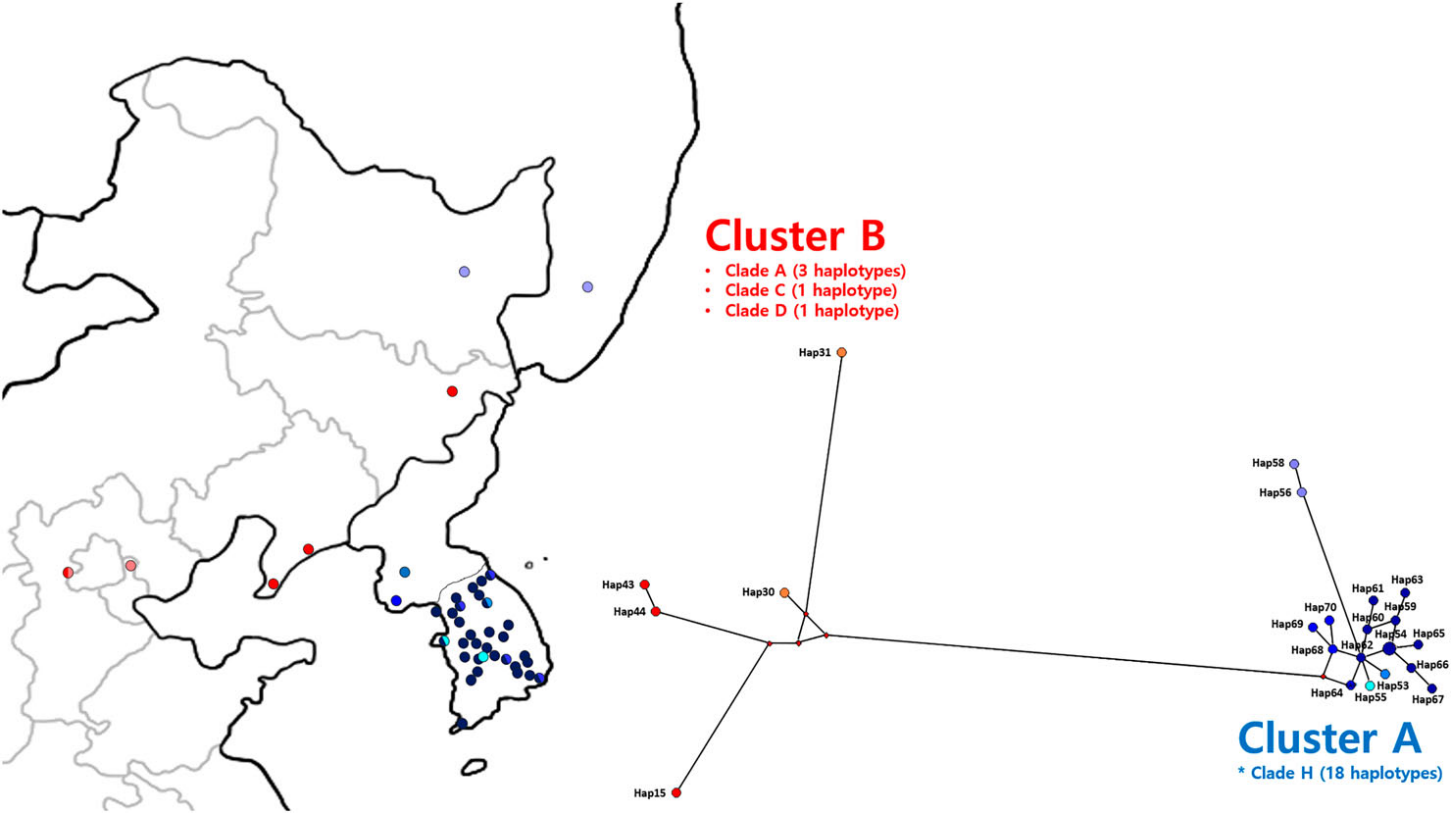
Haplotype distribution network across Northeast Asia, based on the NADH dehydrogenase 2 (*ND2*) dataset. Shades of blue indicate haplotypes in Cluster A, while shades of red indicate haplotypes of Cluster B. Clade information follows Figure 2.

The *ND2*-based genetic network was divided into two major clusters (Figure 4). Cluster A included all haplotypes of Clade H (18 haplotypes), while Cluster B included three clades: Clade A (hap30, hap43, and hap44), Clade C (hap15), and Clade D (hap31). There was clear genetic differentiation between Clusters A and B, despite their close geographic distance. In particular, *B. gargarizans* from Russia and China's Heilongjiang Province were included in Cluster A, although they were closer geographically to some individuals in Cluster B (Jilin and Liaoning Provinces). The mean pairwise difference between the two groups was 0.0503 (0.0451–0.0533) (Table 1).

### 3.3. Divergence dating analysis

**Figure 5.**
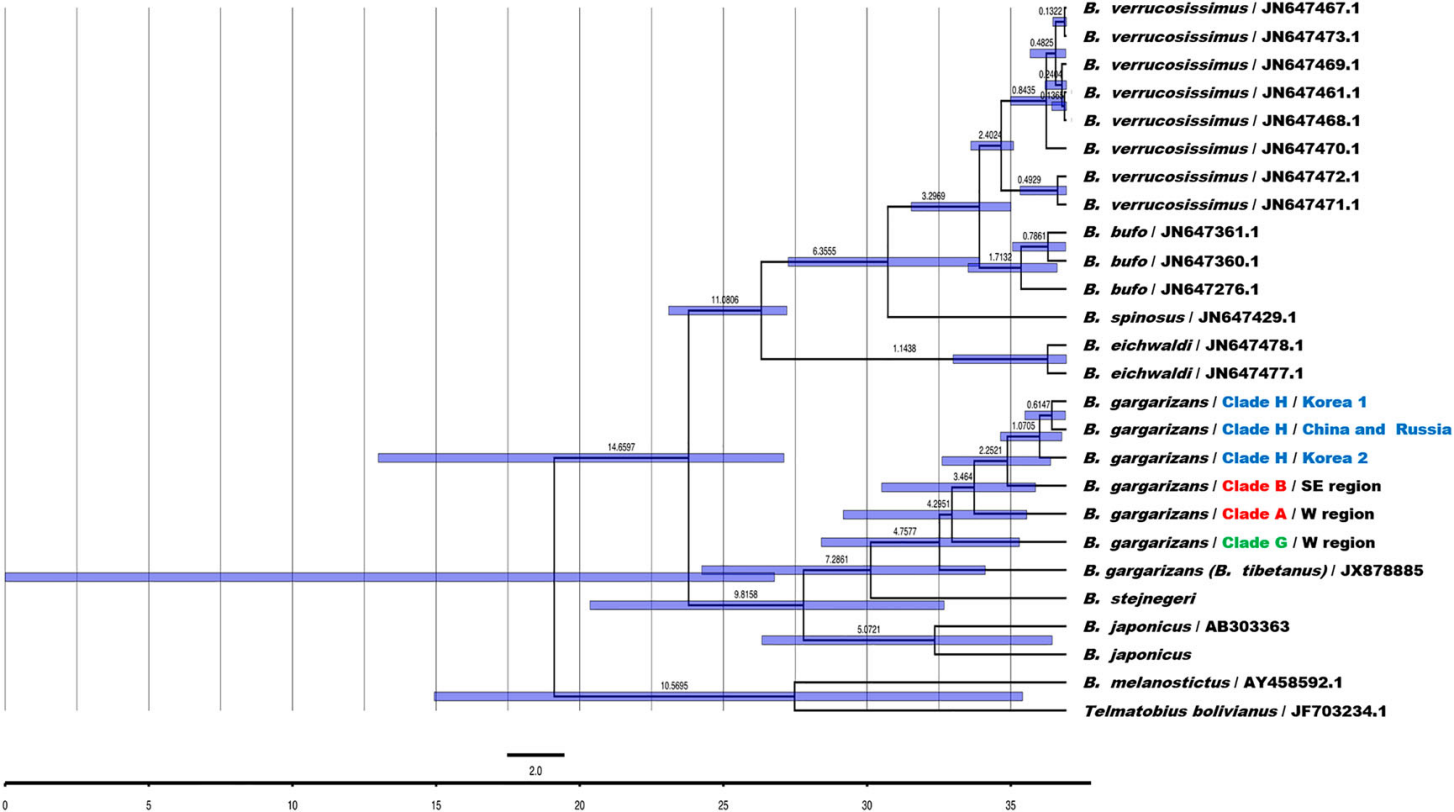
Divergence dating analysis of taxa in the Genus *Bufo* using BEAST. The Cytochrome b (*Cytb*) dataset was used in this analysis. Purple bars indicate the 95% confidence intervals of divergence dates, while the mean is presented along the bars. Dates are indicated in Mega-annum (Ma). Clade information and color follow that in Figure 2.

In general, we recovered broad confidence intervals of the divergence time estimates (Figure 5, Table S6). *Bufo gargarizans* is estimated to have diverged 7.29 Ma (2.83–12.69 Ma), while the major clades within the species are estimated with mean ages of 2.25–4.30 Ma. Our analysis inferred that the divergence pattern of *B. gargarizans* is from West (mean ages 4.76, 4.30 Ma) to Southeast (3.46 Ma) in the Pliocene, followed by Northeast (2.25 Ma) in the early Pleistocene. Divergence within Clade H occurred during the Holocene (1.07, 0.61 Ma).

## 4. Discussion

### 4.1. Genetic structure of B. gargarizans

We contribute to understanding the evolutionary history of *B. gargarizans* by combining broad geographic sampling with a multilocus genetic dataset in a single study. Our mtDNA (*ND2*) data analysis results support *B. gargarizans* being divided into two major groups that are further divided into eight clades (Clades A–H). The genetic status of seven clades (A–G) was identified in a previous study (Zhan and Fu 2011), while an additional clade (H) was newly identified in our study (Figure 2). We uncovered weak phylogeographic pattern for *B. gargarizans*, where the genetic differentiation did not strongly match any geographic pattern. As Zhan and Fu (2011) suggested, for such a genetic pattern to appear, the geographic distribution of *B. gargarizans* would have expanded rapidly after genetic differentiation occurred.

We propose two hypotheses regarding the weak phylogeographic pattern of *B. gargarizans* related to their adaptations to environmental stressors. First, *B. gargarizans* is large-bodied and has a dry, tough skin, allowing it to survive in xeric conditions and to disperse long distances across land relative to other amphibian species. These features of *B. gargarizans* likely played a role in its wide distribution across China. If the range expansion involved many individuals and occurred soon after genetic differentiation, the observed undifferentiated phylogeographic pattern could result.

Second, we suggest that anthropogenic effects contributed to the lack of a clear phylogeographic pattern in *B. gargarizans*. In China, a traditional Chinese medicine (hua chan su, 華蟾素) extracted from skin secretions of toads (including *B. gargarizans*) has been used as medicine for thousands of years (Su and Nu 2001; Meng et al. 2012; Cheng et al. 2019). A current online search for toad farms identifies locations operating in various regions in China (Table S7). Any escape or release of translocated individuals, followed by reproduction with native individuals would contribute to obscuring phylogeographic patterns.

### 4.2. Northeast Asian B. gargarizans

Our study confirmed the presence of two major genetic clusters in Northeast Asia (Figure 4). Cluster A includes individuals exclusive to Northeast Asia (Clade H), while Cluster B includes individuals from across China (Clades A, C, and D) (Figure 2). Our multilocus haplotype network suggests that these two clusters have different origins, with Cluster A likely originating from southeast China, and Cluster B from western and central China (Figure 3). The genetic break between these two clusters seems to occur somewhere between eastern (Heilongjiang Province) and western (Liaoning and Jilin Provinces) regions of Northeast China (Fu et al. 2005; Hu et al. 2007; Tong and Wo 2017). A similar pattern was found in the study of another widespread frog species (*P. nigromaculatus*) – a significant subdivision between Northeast China and other regions of Mainland China (Zhang et al. 2008).

Plant communities also mirror this pattern – mixed conifer-hardwood forest (Heilongjiang and Eastern Jilin Province), steppe (Western Jilin province and Inner Mongolia Autonomous Region), and deciduous forest (Liaoning and Hebei Provinces and Beijing) (Liu 1988; Stebich et al. 2009). Zhang et al. (2008) suggested this genetic pattern was the result of two independent refugia during the last interglacial period in the late Pleistocene. As the divergence of the major *B. gargarizans* groups is older than the Pleistocene, we suggest that the situation is a bit more complex for *B. gargarizans*, with the genetic pattern being shaped by habitat (biogeographic regions), older geologic events (e.g. formation of the Yellow Sea), and multiple glacial refugia. Northeast Asia, although it contains relatively low biodiversity, has had complex geologic and climatic history that deserves additional attention. Finer-scale sampling from Northeast Asia for *B. gargarizans* is needed to sort out the evolutionary history of the species, which will in turn help elucidate the geologic history of the region.

### 4.3. A clade exclusive to Northeast Asia

Our analysis verifies the existence of a clade exclusive to Northeast Asia (Clade H), previously suggested by other studies (Fu et al. 2005; Hu et al. 2007; Borzée et al. 2017). Clade H was strongly supported (bootstrap value = 100) and genetically distinct from other clades (Figure 2). In previous genetic studies, *B. gargarizans* was treated as the *B. gargarizans* complex composed of several clades across a large area without differentiation according to region or altitude (Hu et al. 2007; Zhan and Fu 2011; Borzée et al. 2017). These features made it difficult to understand the evolutionary history of *B. gargarizans*.

The existence of the Clade H suggests new interpretations of the differentiation process of *B. gargarizans* in Northeast Asia. We estimate the divergence time estimate of Clade H to be 2.25 Ma (0.5–4.33 Ma; Figure 5), in the Pliocene and early Pleistocene. During the early Pleistocene (Gelasian age), there were major geological events (landification due to fluctuations in sea level) (He et al. 2015) and dramatic climate change (glacial range expansion). Our multilocus haplotype network infers that the ancestor of Clade H was in southeastern China, as indicated by the mixed membership of Group 7 (SE and FNE regions) (Figure 3).

Previous studies suggested that faunal exchange between China and the Korean Peninsula occurred through the Yellow Sea land bridge at times of low sea levels (Zhang et al. 2016; Du et al. 2019), including *B. gargarizans* (Borzée et al. 2017). After dispersal into Northeast Asia, the subsequent rise of sea level and the expansion of glaciers would have isolated Clade H in a glacial refugium on the Korean Peninsula. A similar pattern of a glacial refugium on the Korean Peninsula was found in other organisms (Lee et al. 2008; Yoshikawa et al. 2008; Zhang et al. 2008; Kim et al. 2013; Borzée et al. 2017; Fong et al. 2020). Although we had limited sampling from North Korea, Heilongjiang Province (China), and the Russian Far East, there is a preliminary pattern indicating that South Korea is relatively diverse, which would support a scenario of range contraction into South Korea during a glacial cycle, followed by a range expansion northward during an interglacial cycle (Figure 4). To verify this hypothesis, additional samples are needed from in the Northeast China, North Korea, and the Russian Far East.

## 5. Conclusion

*Bufo gargarizans* is a genetically diverse species distributed broadly across East and Northeast Asia. Our study uncovers the presence of a new clade restricted to Northeast Asia. We demonstrate the complex genetic pattern of this species, where most of the genetic divergence is not associated with geographic regions. We suggest that this pattern is a result of multiple influences – robust dispersal abilities resulting from ecological characteristics, anthropogenic influence of translocation, geologic activity, and glacial cycles of the Pleistocene. We highlight the complex geologic and climatic history of Northeast Asia and encourage further research to understand its impact on the biodiversity in the region.
